# Determinants of highly-cited green patents: The perspective of network characteristics

**DOI:** 10.1371/journal.pone.0240679

**Published:** 2020-10-30

**Authors:** Kuang-Cheng Chai, Yang Yang, Zhiyong Sui, Ke-Chiun Chang

**Affiliations:** 1 School of Business, Guilin University of Electronic Technology, Guilin, China; 2 School of Economics and Management, Wuhan University, Wuhan, China; Politecnico di Milano, ITALY

## Abstract

With the rise of green technology, green patent value evaluation has become critical for enterprises. In order to explore the influencing factors of green patent value, this study takes the light-emitting diode (LED) industry as an example and then utilizes highly-cited green patents as the proxy of green patent value. The study aims to explore the influencing factors of green patent value with network position and structure from the perspective of network characteristics. The results indicate that out-degree centrality and network constraint have a significantly positive effect on green patent value.

## Introduction

As environmental pollution and resource shortage problems increase, many means have been taken, including the development of green technologies. This refers to technologies/products that use cleaner and more energy-efficient raw materials. Green technologies reflect humanity’s introspection about the consequences of the damage caused to the ecological environment by modern sciences and technologies. Based on the high value of green technologies, green patents have rapidly developed. The R&D of green technologies and patents have created both opportunities and challenges for enterprises. On one hand, the development of emerging green industries has created a huge market for enterprises, which can increase the sale of products and earn more profits. On the other hand, in order for enterprises to meet the national environmental standards proposed by the policy, additional costs are incurred. Therefore, enterprises must upgrade their existing equipment and invest more in R&D to produce more environmentally-friendly products in response to these changes.

Although green patents are receiving greater attention, so far there has been relatively little research into the value of these patents, and most research mainly focuses on the institutional level. Some researchers have made progress by establishing evaluation indicators, constructing models, and conducting empirical analysis in the area of patent value [1–5]. In addition to using patent characteristics, such as the number of forward and/or backward citations, and claims to measure a patent value, researchers have also discovered some professional and comprehensive indicators, such as patent innovation and maturity of technology [6, 7], the technological importance [8], innovation-basic research linkage, technological scope [9], and legal breadth of the protection [10, 11]. However, they only considered patent characteristics separately and ignored the role patents play in the process of technological evolution. In review of the development of technologies from the perspective of social network, constructs a patent citation network based on the analysis of the mutual-citation network relationship in the network among patents contributes to explaining the connection of technologies and the evolution trend of technologies [12].

Except patent itself characteristics which may impact its value, the network position and structure of the patent in the patent citation network will also affect its value. Patents that play an important role in other citation relationships indicate that highly-cited green patents have a higher probability of obtaining and controlling technologies and knowledge, and have a greater impact on subsequent green patents. In other words, the significance of a green patent value is usually determined by the importance of the role it plays in the technical-knowledge flow process; a highly-cited green patent that pushes forward the entire technical knowledge evolution has a higher value than other green patents. Therefore, this study adopts the method of network analysis, using patents as nodes and connecting the flow of technical knowledge between patents to build a patent citation network, which measures the network position and network structure of patents. Bessen [4] finds that highly-cited patents are more valuable, besides, highly-cited patents also contained important technological advances [13, 14]. Therefore, this study used four indicators of network characteristic: degree centrality, closeness centrality, eigenvector centrality, and network structure, to explore the relationship between network characteristic and highly-cited patents to fill this research gap. This study could help enterprises to evaluate green patents, to construct patent pools more efficiently, and to establish better competitive strategies.

## Literature review and hypothesis development

### Social network analysis

Social network analysis (SNA) is the method of studying social relationships and structures, mainly focusing on the internal network between different social actors [15]. Social network analysis was developed in the 1930s and was used to analyze the relationships between people. It rapidly developed and was combined with mathematics, statistics, computer science, and other disciplines to become an interdisciplinary analytical method.

A social network is comprised of nodes and sociological ties. Nodes imply actors, which may be the individual, team, enterprise, patent, or other social organizations. Ties imply the relationships between nodes, and these ties are described using matrices and graphs. The distribution and characteristics of the relationships in a social network can be analyzed using a matrix, and the structure between the nodes and the overall properties of the network can be observed using a network graph that connects the actors.

**Fig 1:**
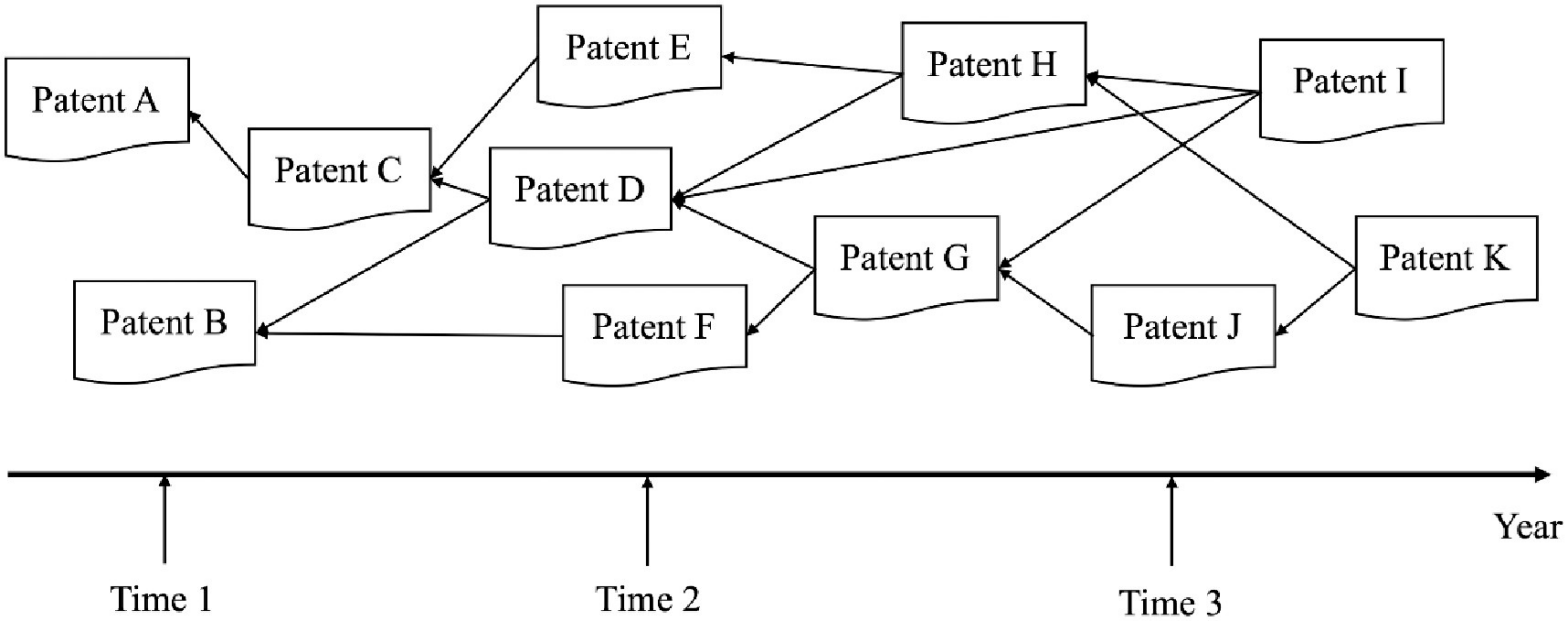
The network of patent citations.

This study considers the actors in the social network as patents, then the relationship between the patents will constitute a technology network in which one node represents a patent. Fig 1 shows the network of patent citations, where A–L stands for patents and arrows indicate citation relationships. The citation relationship indicates that a new patent is related to another technological innovation.

Since Yoon and Park [12] applied network analysis to patent research, an increasing number of scholars has begun to build patent citation networks for analysis [16–24]. For example, Gress [16] highlighted that the internal and external references in patent citation networks can reflect the technical dependency and related contributions between the patent groups. Kim and Song [17] and Chang et al. [24] introduced a network analysis to analyze the litigation relationships between smartphone manufacturers and uncovered four key categories. Lee et al. [18] studied the conductive polymer nanocomposite industry and constructed a patented network diagram and discussed the evolution of the technology by calculating and analyzing the relative distance and network location of the patent within the network. Hsu and Lin [23] established a patent citation network through 118 biomass technology patents, and proposed a method for determining core or key patents.

Bruck et al. [19] asserted that patent citation networks can be considered as a complex system that evolves over time. Huang et al. [21] applied network analysis to explore the relationships between patent value and network centrality and network position. Huang et al. [21] used litigated patent as the proxy variable for patent value. However, patent litigation is costly and time-consuming. If a company loses the lawsuit, it may mean the loss exclusive access to the patent technology, leading to legal fees and reduced profitability. Therefore, many patent litigation cases will choose out of court settlements, in that case, using patent litigation as a proxy variable will underestimate the value of patents. There, in this work, we focus on the highly-cited green patent, using a network analysis to analyze the influencing factors of green patent value.

Social network, which focuses on network structure and participant’s position in the network, not only explores the different positions of participants in the network and the network structure formed by the relationship between two or more participants and the third party, but also analyzes the characteristics, generation and evolution of the structure. To be specific, the analysis social network analysis is mainly based on the following two theories.

1. Social capital theory: Organizations or individuals in society acquire resources and create social capital by their interconnections. And it is the complex network consisted of such direct or indirect interconnections that promotes the flow of various social capitals in the entire network. Secondly, relations in the social network are not absolutely equal. The quantity of social capital got by an organization or an individual determines its position in the social network. Organizations or individuals in the center of the network usually have more connections with the outside world, which is beneficial for them to acquire more resources and have more social capitals. Finally, the quantity of social capital may influence organizations’ or individuals’ selection of new relations in the outside world. In other words, to get greater competitive advantages, organizations or individuals usually prefer organizations or individuals in the center of the network.
2. Structural hole theory: Structural hole particularly probes into the influence of network structure and their position in the network on network participants. According to structural hole theory, a relatively closed structure makes the network have more homogeneous information, and participants’ repetitive information cannot wide the gap between them. On the contrary, in the open network structure, structural holes enable participants in the position of holes to control the flow of information and resources in the network by connecting diverse nodes, by which participants can improve their competitive advantages. Therefore, the following hypotheses in this study are based on social capital theory and structural hole theory.

### Network centrality and patent value

Network centrality is the focus of social network research, and it is an important structural location indicator [25]. In sociological research, centrality is often used to measure the importance of the individual, the status of superiority, and the social prestige. Freeman [26] argues that the central place in the social network reveals the rights and status of an individual in a group, which means that the more an actor is at the center of the network, the greater is his or her influence. By applying network centrality to the patent citation network, the patent can be used to measure the position of the patent and to identify the higher patent value in the whole patent network.

#### Degree centrality

Degree centrality evaluates the positions or superiorities of nodes in the social network, reflecting the direct influence of actors in the network. If an actor has direct ties with many actors in a social network, then the actor is at the central position and has widespread influence. In a directed graph, the degree of centrality can be divided into in-degree centrality and out-degree centrality according to the direction of the relationship or interaction between the actor and other actors. In-degree centrality refers to the number of other actors to direct a relationship toward the central actor, while out-degree centrality refers to the number of relationships the central actor directs towards others in the network.

As the patent citation network is an ordered network, according to the different citation orientation between patents, forward citation can be regarded as the out-degree centrality, while backward citation can be regarded as the in-degree centrality. According to the previous section, the number of patents citation as the evaluated indicator is an overlap with the in-degree centrality of patents, so we only consider the out-degree and ignore the in-degree centrality.

As for patents, the out-degree centrality reflects the references and inheritances from other patents, and this indicator also shows the degree of this patent’s dependence on other sciences and technologies. In this rapidly developing modern society, most patents are developed and improved based on already-existing skills or technology. From this perspective, if a patent cites technology already in use and that the technology being patented is mainly an improvement of existing and mature skills or technology. Therefore, if a patent cites more previous patents, it is likely related to a large number of comparatively mature technologies, which means that the patent can integrate existing knowledge and technology better and that its technical coverage is broader that of other patents [27–29]. Allison et al. [30] pointed out that the more valuable patents usually cited more advanced technology and are more easily cited by other patents. Thus, the higher the value of a patent, the more patents it will cite, meaning that the patent has higher out-degree centrality. Therefore, we present the first hypothesis:

***H1*: *Compared to little-cited green patents*, *highly-cited green patents have higher out-degree centrality***.

#### Closeness centrality

Closeness centrality is the sum of the shortest distance from a node to all other nodes in the network. Compared to the out-degree centrality, it represents the overall centrality of a node instead of its partial centrality. Closeness centrality evaluates the distance between a node and other nodes; the shorter the distance, the more easily each of the nodes can reach other nodes. In a social network, if an actor is connected to many other actors through a relatively short path, the actor has a high degree of nearness to the center, making the actor the center of the network.

Freeman [26] stated that if all paths through which an actor is connected to other nodes are short—meaning that the actor is close to many other actors—the actor is less dependent on other actors in the process of information transfer. Actors occupying non-core positions must transfer information through lots of other actors. The actors occupying the near-center position, however, are more efficient in communicating information throughout the network because of their short path to other actors. Such actors also tend to act in key roles, which can quickly reflect in the process of problem resolution. Thus, closeness centrality is the primary measure of the ability of the actor to not be controlled by other actors and a measure of the efficiency of information transmission between actors.

Similar to degree centrality, the closeness centrality of directed graphs can be divided into that of in-closeness centrality and out-closeness centrality. Closeness centrality is a passive approach of the actor and refers to the sum of the shortest distances required by other actors to contact or cite actors. In contrast, out-closeness centrality refers to the sum of the shortest distance the actor wants to associate with or quote from other actors, reflecting the active approach of the actor. This paper addresses only the outward-near centrality of patents, the sum of the shortest distances required for a patent to be directly or indirectly cited by all other patents. When the outward orientation of the patent is close to the central degree, the patent can efficiently absorb information and knowledge found in the previous patent citation network and acquire technical knowledge or information from other fields. In contrast, the inventor of the patent may more effectively cite previous patents to develop and improve upon extensive reference to other patents, and the patent may therefore contain higher technical content and be more valuable than other patents. Thus, we propose hypothesis 2:

***H2*: *Compared to little-cited green patents*, *highly-cited green patents have higher out-closeness centrality***.

#### Eigenvector centrality

The out-degree centrality simply measures the number of direct connections to other nodes. The eigenvector centrality reflects the importance of a certain node in a network. Its key idea is that the importance of a node is determined by both the number of its connections with other nodes and the importance of the nodes with which it is connected.

As for the patent network, eigenvector centrality weighs the most significant core patent for this index and measures the patent and its contact patents’ centrality at the same time. If one patent has relationships with many other high-centrality patents in the citation network, then the patent is important in this particular network. In contrast, a patent may be at the center of a local patent citation network but not the entire network if the patent is connected to a number of non-central patents.

We can assume that actors that connect to an actor with high eigenvector centrality are always connected to many other actors. Thus, if a patent has high eigenvector centrality, the patents that cite or are cited by this patent also have high centrality. Therefore, we can say that a patent with a higher eigenvector is closer to the core position of the patent citation network. The patent can influence many patents through direct or indirect contact in the entire citation network. It can be said that the greater the influence of such a patent, the higher its value. Therefore, hypothesis 3 is as follows:

***H3: Compared to little-cited green patents, highly-cited green patents have higher eigenvector centrality***.

### Network structure and patent value

In addition to the centralities of specific nodes in the network, the network structure plays an important role in social network analysis. Research has often divided the network structure into open networks and closed networks. Open structure can be linked to different groups and can control the flow of information through the intermediary position. At the center of this perspective is Burt’s structural hole theory [31, 32]. In Burt’s theory, structural holes are some of the gaps in network relationships; that is, there is an indirect rather than a direct link between some actors in network relationships and other actors. Moreover, structures with gaps between the actions can contain less redundant information, and therefore, structural holes can affect and improve the efficiency of the entire network of information exchange. In addition, the advantages of this network structure also derive from the intermediary opportunities created by information exchange. In an open network structure, the actors can establish contact with groups that are not associated with each other and obtain more resource advantage by controlling the information channel [22].

In contrast to open networks, closed network structures can facilitate the flow of information within the network and help foster the trust between internal actors, which is the positive effect of network closure. Specifically, the actors in this closed network structure have a high joint density, so the cohesion of the network will be very strong. If there is abnormal information or abnormal behavior in the entire network, each actor can quickly respond, and the abnormal information or behavior can be excluded from the network, benefiting all actors.

There are two metrics for measuring the degree of network closure: network constraints and network effect size [31, 32]. The network effect size is used to measure the overall influence of the structure-hole actor, which is defined as the sum of the non-redundant links in the network structure. The network constraint measures the degree of dependence of an actor on the other actors in the network and the amount of redundant information contained in the relationship. In this study, we use the constraint to measure the network structure of a specific node. The larger of the network constraint, the less non-redundant links there are in the network. Therefore, we present the following hypothesis:

***H4*: *Compared to little-cited green patents*, *highly-cited green patents have higher network constraint***.

## Methodology and measurement

### Sample and data collection

As an important part of green patents, light-emitting diodes (LEDs) embody the concept of green energy and are at the forefront of the global electronics industry. The characteristics of LEDs include low power consumption, long life, fast response time, and the absence of pollution. These characteristics make it extremely important in the field of semiconductor lighting. Optoelectronic technology represented by LED is the most promising industry technology of the 21st century. Therefore, this study takes the light-emitting diode industry as an example. This study uses data concerning LED patents in the US found in the Thomson Innovation database as of May 2011, including three technical areas: LED epitaxial growth, chip fabrication, and chip packaging technology. Finally, a total of 4,650 patents were found. We converted a citation data of 4,650 patents into the adjacency matrix {*M*_*ij*_}_4650*X*4650_, *M*_*ij*_ represents whether patent *i* cites patent *j*, If cites, it takes a value of 1, and otherwise, it takes a value of 0, which were then used for establishing a patent citation network for the green patents (LEDs).

Traditional indicators of patent value evaluation like patent forward citation frequency have their own advantages and disadvantages. However, the major aspect of evaluation lies in the balance between quality and quantity. If patent forward citation frequency is taken as the only indicator, people may hit a bottleneck as quantity may become the single focus. And because it is difficult to evaluate both quality and quantity at the same time, this problem has not been solved yet until Hirsch [33] put forward H-index. The reason for Hirsch to propose H-index was because there were deficiencies in the traditional evaluation indicators and highly-cited patent was not brought to the forefront. And actually, highly-cited patent is the most important aspect in the evaluation of scientific research.

**Fig 2:**
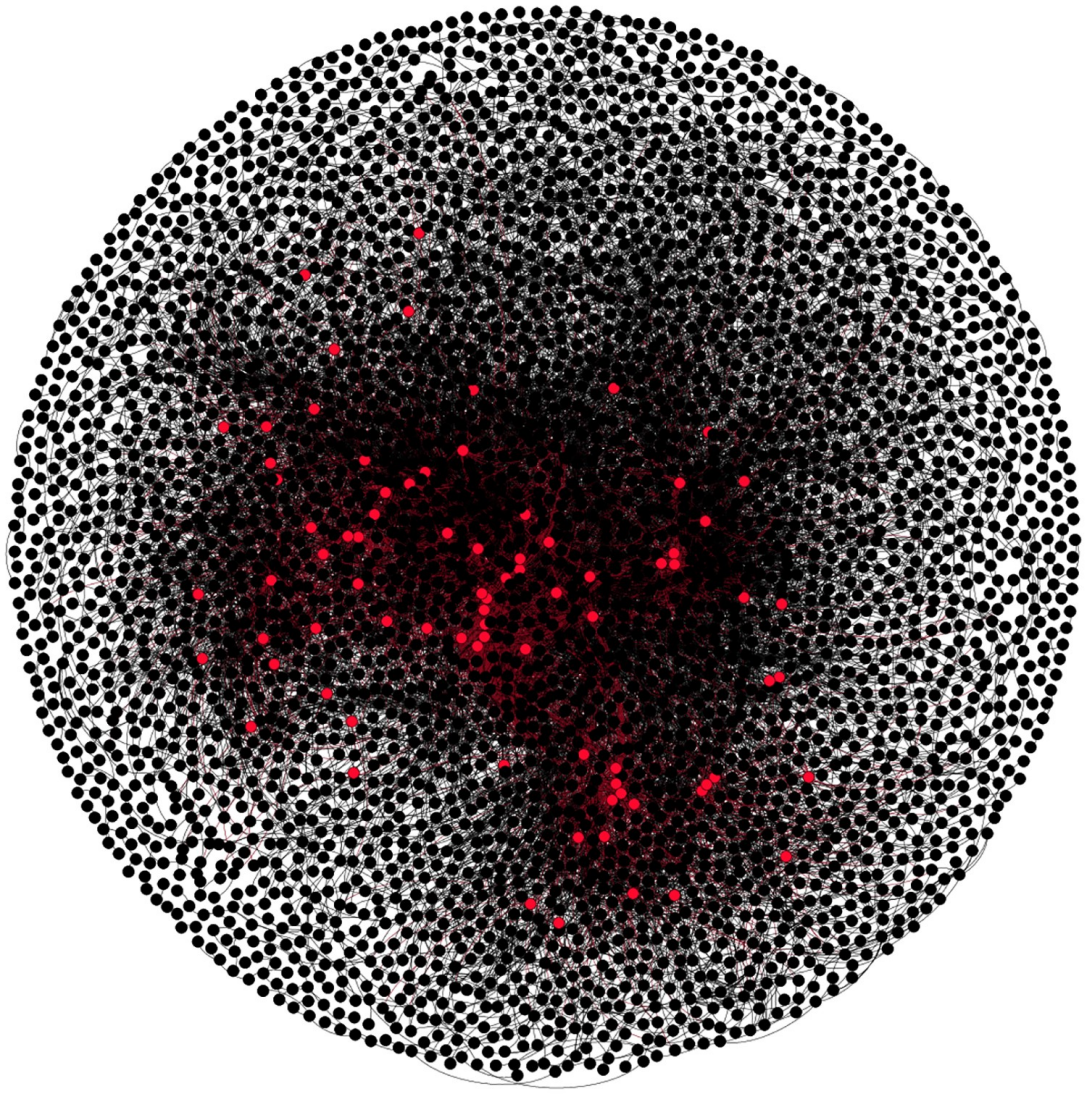
Patent citation network for the green patents (LEDs). Note: Red dots represent highly-cited green patents.

This study uses the H-index to evaluate the value of green patents. The H-index was originally used to analyze the individual academic achievements of scientists and was a new indicator proposed by Hirsch in 2005 [33]. Hirsh defined the H-index as: In the N papers published by the scientists, the maximum number of papers is quoted at least h times. Compared with a traditional evaluation index, the H-index takes both the number and the quality of scientists into account. Further, Guan and Gao [34] showed that the H-index can effectively evaluate the technical importance and influence of the patent assignee. Fig 2 shows the patent citation network for the green patents that includes patent cited by and patent citing. The red dots represent the highly-cited green patents.

Since then, the H-index has been widely used to assess the value of patents [35, 36]. According to the H-index, green patent is divided into two types: highly-cited green patents (the number of forward citations above the H-index) and little-cited green patents (the number of forward citations below the H-index). After the initial calculation of LED patents, the H-index is 105; that is, of the 4,650 green patents, 105 green patents are cited more times than their serial number. Such a green patent is defined as a highly-cited green patent. We then selected the related patents based on the principle of highly-cited green patent and little-cited green patent ratio of 1:2 for data screening.

Due to differences existed in between high-cited patent and little-cited patent characteristics, in order to avoid endogeneity problem caused by sample-selection bias, this paper uses 6 matching variables to find little-cited patent samples similar to high-cited patent samples, including that the patent application is the same or close to the year of authorization; same technology field; identical or similar number of claims, inventors and patent families. However, there are 20 highly-cited green patents that cannot accurately match the relevant little-cited green patents. Such non-matching highly-cited green patents were excluded, and a total of 255 green patents were examined in this study, including 85 highly-cited green patents and 170 little cited green patents (S1 Data).

### Measurement

The definitions and measurements of the variables were further defined as follows.

Dependent variable: Green patent value: We used the H-index of the green patent to divide patents into highly-cited and little cited green patents. “highly-cited green patent” is adopted as the proxy variable for “Green patent value”. The dependent variable is a categorical variable which is coded 1 if the patent has highly-cited green patents and 0 if the patent has little cited green patents.

Independent variables: Out-degree centrality (OutDegree): the number of times that patent *i* cites other patents or the number of patent *i*’s backward citations; defined by *d*(*i*) = ∑_*j*_ *x*_*ij*_, if patent *i* cites patent *j*, *x*_*ij*_ = 1.

Out-closeness centrality (OutCloseness) is the sum of the shortest distances from node *i* to other reachable nodes; defined by $c(n_{i}){=[\sum_{j=1}^{n}d(n_{i},n_{j})]}^{-1}$, *d*(*n*_*i*_, *n*_*j*_) is the shortest distance from node *i* to node *j*.

Eigenvector centrality (EigenVector) is the central index of node *i* after considering the centrality of other nodes connected to *i*, and it measures the degree of influence of the other nodes on node *i*. The eigenvector centrality is calculated as $e(i)=\lambda^{-1}\sum_{j=1}^{n}a_{ij}e_{j}$, *a*_*ij*_ represents whether node *i* and its adjacent matrix (*i*, *j*) are connected. If connected, it takes a value of 1, and otherwise, it takes a value of 0. λ is the eigenvalue of the adjacent matrix, and *e*_*j*_ is the eigenvector corresponding to each eigenvalue.

Network constraint (Constraint) is often used to measure the node’s impact on the entire network, reflecting the degree of non-redundant information in the network. The constraint of node *i* is calculated as *C*_*i*_ = ∑_*j*_(*P*_*ij*_ + ∑_*q*≠*i*,*q*≠*j*_ *P*_*iq*_*P*_*qj*_)^2^, where *P*_*ij*_ is the proportion of patent *i*’s invested in connection with contact *j*, and *P*_*iq*_*P*_*qj*_ is the sum of patent *i*’s indirect invested in connection with contact *j* via all *q*.

## Results

### Descriptive statistics

Descriptive statistics of this study are shown in Table 1, including minimum, maximum, mean and standard deviation.

**Table 1:** Descriptive statistics.

Variables	Mean	S. D.	Min.	Max.
HighCitedPat	0.33	0.47	0	1
OutDegree	15.09	23.17	0	134
OutCloseness	0.07	0.01	0.07	0.09
EigenVector	0.02	0.03	0	0.17
Constraint	0.17	0.20	0	1.13

### Results of logistic regression analysis

In this study, a logistic regression model was established with high citation green patent as the dependent variable, OutDegree, OutCloseness, EigenVector, and Constraint as the independent variables. The results are shown in Table 2.

**Table 2:** Results of logistic regression analysis.

Variables	Logit model	Marginal effects
Intercept	5.29	
	(4.65)	
OutDegree	0.17**	0.04**
	(0.03)	(0.01)
OutCloseness	-116.51	-26.55
	(65.70)	(15.35)
EigenVector	-32.08	-7.31
	(17.82)	(4.03)
Constraint	2.76**	0.63**
	(0.90)	(0.20)
-2 Log Likelihood	-190.05	
Prob > χ2	0.000	

Note:

**p<0.01,

*p<0.05, Standard errors are reported in parentheses.

As shown in Table 2, the coefficient of OutDegree is 0.17 at the 1% significance level. The Constraint coefficient is 2.78 at the 1% significance level. These mean that the OutDegree and Constraint have a significant positive effect on patent value. Therefore, this study verifies the correctness of hypothesis 1 and hypothesis 4. However, OutCloseness and EigenVector have no significant effect on patent value, so hypothesis 2 and hypothesis 3 are not verified in this study. Besides, the collinearity test show that the variance inflation factor (VIF) is less than 10; there is therefore no multiple collinearity among independent variables.

The second column in Table 2 is the estimated marginal effect of network characteristic variables on the probability of highly-cited green patent under the mean value of explanatory variables. The marginal effect of these variables indicates that if their values increase by one standard deviation, it will cause a change in probability. The average of the outcome variables is shown at the bottom of the table. The results show that the network characteristics have an important influence on the probability of highly-cited green patent, and the marginal effect of several patents (OutDegree and Constraint) is significantly different from zero. This study mainly studies the influence of network characteristics. The Constraint has a significant positive partial effect on the probability of highly cited green patent. For every additional standard deviation, the probability of highly cited green patents increased by 63%. Among the network characteristics, the influence of OutDegree on the probability of highly-cited green patent ranked second. As expected, the effect was positive. Each increase in the standard deviation significantly increased the probability of highly cited green patents by 4%.

Errors in measuring network characteristics might also generate endogeneity. This study uses degree centrality (out-degree, out-closeness and eigenvector) to quantify the network position and uses constraints to measure the network structure, while in additional analyses this study uses the variable substitution method to improve the robustness of the result. This paper adjusts network characteristics by the mean in consideration of the impact of difference technology fields on network characteristics. To be specific, the study subtracts the mean from the value of each network characteristic and finally gets the adjusted indicators of network characteristics. The results in Table 3 are consistent with the results in Table 2.

**Table 3:** Results of robustness check.

Variables	Logit model
Intercept	-2.13**
	(0.26)
Adj-OutDegree	0.18**
	(0.03)
Adj-OutCloseness	-121.01
	(65.86)
Adj-EigenVector	-32.65
	(18.32)
Adj-Constraint	2.74**
	(0.89)
-2 Log Likelihood	-189.88
Prob > χ2	0.000

Note:

**p<0.01,

*p<0.05, Standard errors are reported in parentheses.

## Conclusion and discussion

This study aims to find out which network characteristic are more important and the relationship between highly cited patents. This study divides the green patents into highly-cited and little-cited green patents according to the H-index and calculate the network centrality indexes by constructing the patent citation network to analyze the green patent value. The results show that there are significant differences between highly-cited and little-cited green patents. Combining the results with network analysis, it is found that there is a significant positive correlation between the out-degree centrality of green patents and the green patent value. The higher the degree of patent expulsion is, the higher the green patent value. Additionally, constraint has a significant positive impact on green patent value. The larger the network constraint, the greater the green patent value. Out-closeness centrality and eigenvector centrality do not have a significant impact on green patent value.

This study can be used as a reference for enterprises to identify key green patents when evaluating green patents. It is important for practitioners in the green technology industry to understand this relationship. Therefore, when assessing the value of green patents, enterprises can construct a patent citation network and calculate the relevant network index of the specific green patent to determine its value and to choose a relatively high-value patent for building a favorable patent portfolio, which can achieve cost-maximization of technological innovation and sustained competitive advantages. At the same time, enterprises can also build a citation network to analyze the history and development trends of technological fields and to identify the highly-cited green patents in the development, and the positions in which the companies themselves and their opponents are located. Enterprises can then analyze and compare the layout strategies of themselves and their opponents to formulate appropriate competition and cooperation strategies.

This study could help enterprises to construct patent pools more efficiently. In the process of green patent applications, in addition to the characteristics of the patent itself, the enterprise should also place patents in the all-patent network to consider their value. Due to the positive correlation between constraint of the patent network and green patent value, the enterprise can apply green patents on the basis of extensive reference to existing technology and strengthen the technical cooperation within the same field to more redundant information across the entire network.

**Fig 3:**
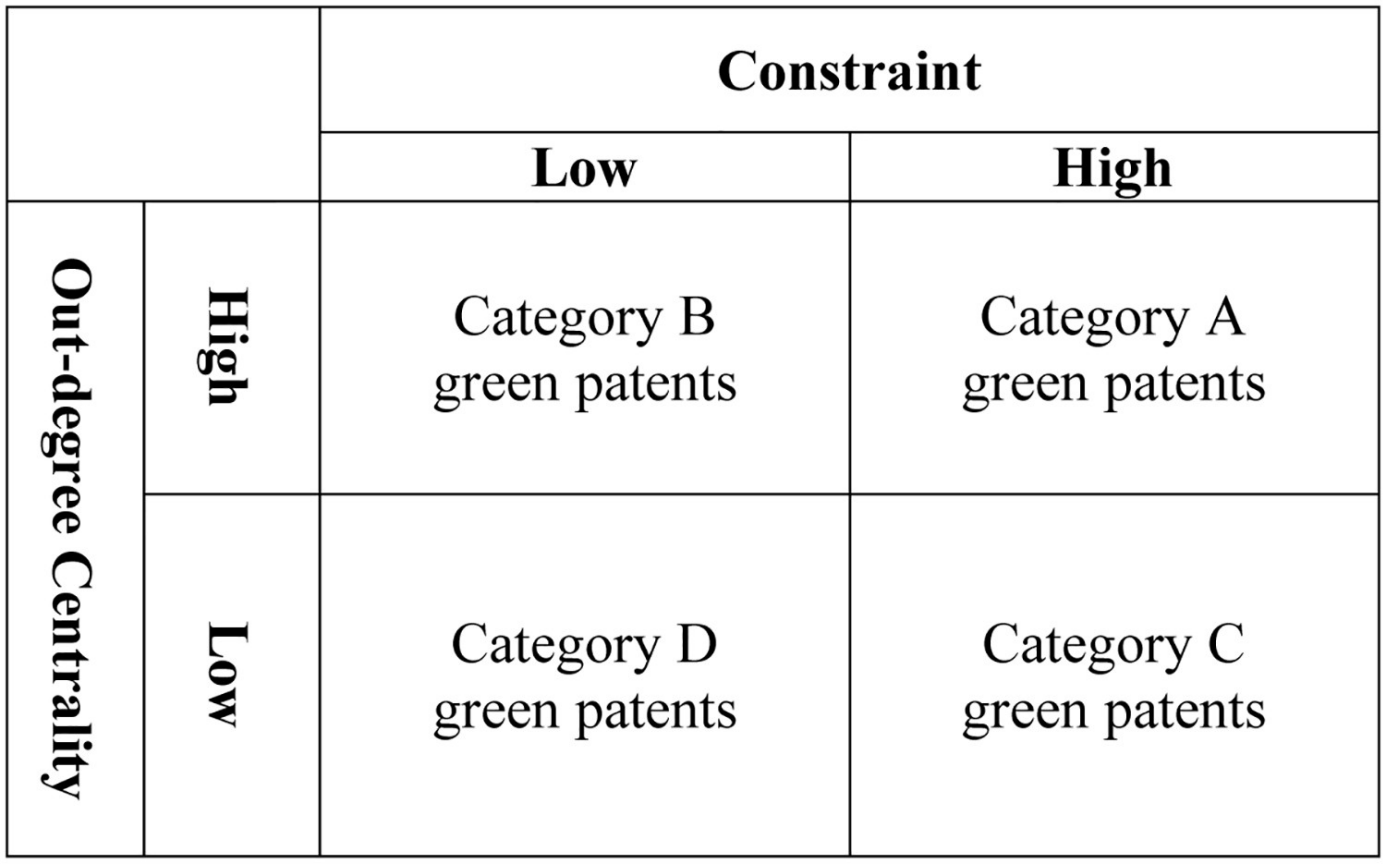
The classification for the green patent.

By adopting an of out-degree centrality index of green patents and network constraint, we divided green patents into four categories, as shown in Fig 1. As can be seen in Fig 3, category A green patents have higher out-degree centrality and a greater network constraint. This is the highest value type of green paten and enterprises should pay attention to licensing or the possibility of a law suit being filed. Category B green patents have higher out-degree centrality and a smaller network constraint. Category C green patents have lower out-degree centrality and a greater network constraint. These two kinds of patent should be monitored to observe if they are profitable. Category D green patents have the lowest value and their applications are costly for enterprises. Thus, enterprises are recommended not to apply for Category D green patents. At last, this study hopes that the results can be helpful to managers, researchers or the government, and provide references for relevant research and future research.

## Supporting information

S1 Data The raw data of this paper. (XLSX)
